# Supplementary figures and images for: Role of Erythropoietin Receptor Signaling in Macrophages or Choroidal Endothelial Cells in Choroidal Neovascularization
Source: Biomedicines. 2022 Jul 9;10(7):1655. doi: 10.3390/biomedicines10071655 (PMC9312702; doi:10.3390/biomedicines10071655)

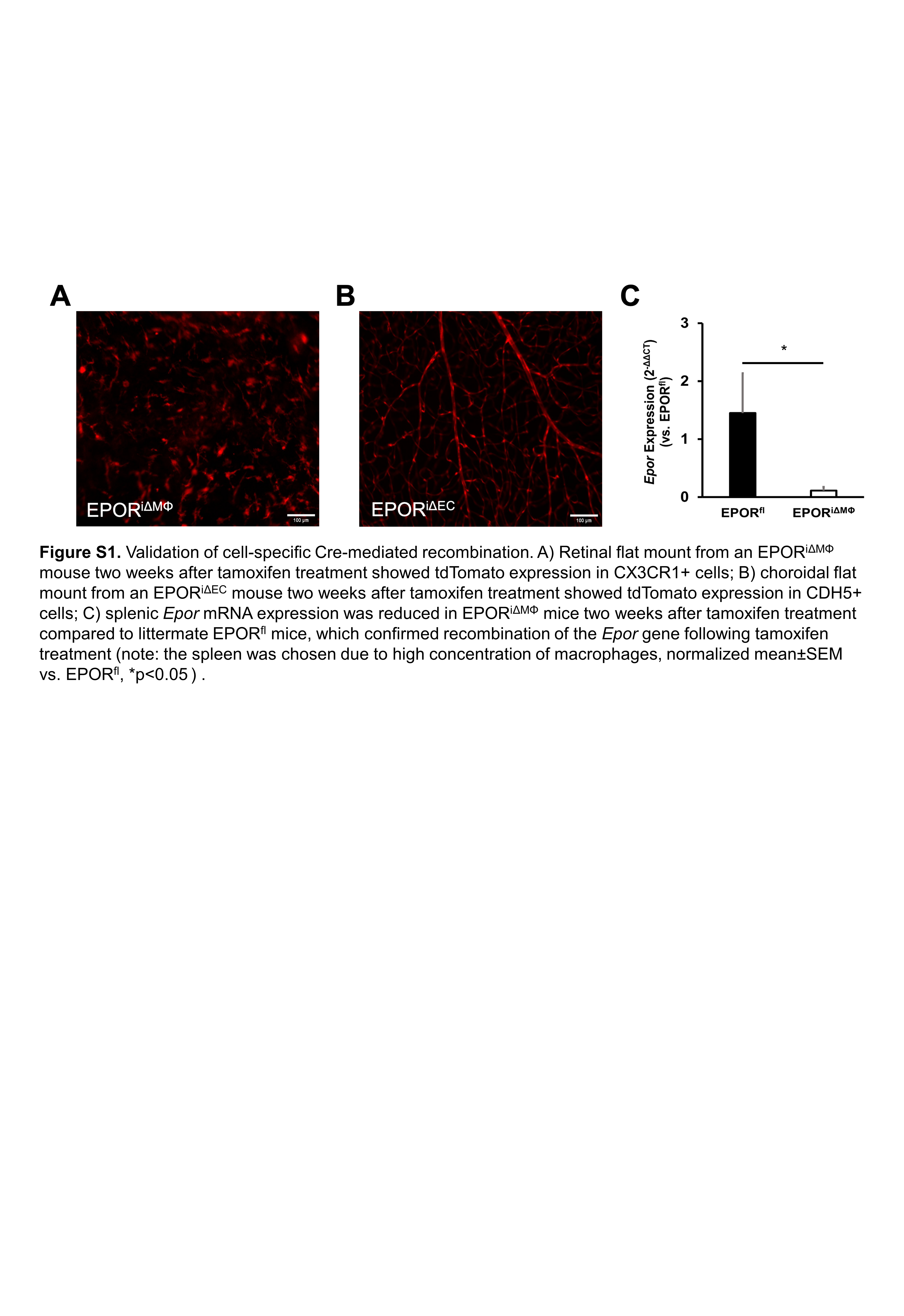

Supplement: Supplementary file 1 [file biomedicines-10-01655-s001.zip › Figure S1.PNG]
